# A Case, and Its Perplexities

**Published:** 1871-11

**Authors:** H. Scott


### A CASE, AND ITS PERPLEXITIES.

BY H. SCOTT.

Messrs. Editors: I give you a concise statement of a case that disturbed my equanimity to some extent. I do not choose to append any remarks, and only state the case with the hope that I may be able to help some one who may chance to be placed in similar circumstances.

Recently a robust farmer, of middle age, called to consult me about a swelling in his mouth, which he said was very painful. I found a phlegmonous inflammation of subacute character, opposite the first right upper molar, and extending up to the median line. The tumefaction was considerable, and to the touch was found to be very tense. It had been induced by the condition of the palatine root, which was of unusual length, and was denuded of its natural covering quite up to its apex. It had been coming on slowly, he said, for several days. The tooth stood the sounding hammer, showing that there was no periosteal inflammation about the roots; but notwithstanding, because the restoration of the soft and hard involucres of the root would be impossible, I advised the immediate removal of the tooth. He would rather keep the tooth if the inflammation could be dissipated, the more especially as he dreaded the pain of its removal. The inflammation had not risen to the degree when pustulation was likely to terminate it; and as there was little chance for encouraging the formation of abscess by emolients, on account of the position, I told him we must direct our attention to the cure by the first intention. I therefore truncated the parts by free incisions with a very sharp bistoury, and sent him away to call again in two days. He came at the time and the inflammation was still of the same chronic character, though the tension of the parts was less, and the tumefaction perceptibly subsiding. I told him he was doing as well as I had expected, and prescribed a solution of sacharum plumbi, to be applied with wet rags, and held for a time in contact several times during the day and night. This treatment has always done good in such cases under my hands, changing the parts to a normal condition.

At the end of four days he presented himself again, to say that he was suffering more than ever. I found the entire buccal cavity, clear down to the crest of the soft palate of a fiery red color, and the mucous membrane thickly studded with unsightly granulations. I said, sir, you have not been using the sugar of lead wash at all; you have been using local irritants, and you are in a bad fix. He expressed alarm, and wanted to know if there was danger that it would kill him. I replied that I thought not, if he would stop the use of his stimulants; but I would not prescribe under the circumstances, and he went away. He had been plying, under other advice, carbolic acid and potash washes alternately. I told him I thought he would have white ulcers in his mouth by the next day.

